# Cognitive factors on the performance of group decision-making: a behavioral and eye-tracking study

**DOI:** 10.3389/fnhum.2025.1551447

**Published:** 2025-03-17

**Authors:** Cheng Kexin, Jiang Zuhua, Yang Jiapeng

**Affiliations:** School of Mechanical Engineering, Shanghai Jiao Tong University, Shanghai, China

**Keywords:** interdisciplinary, group decision-making, eye-tracking, engineering product design, cognitive factors

## Abstract

**Introduction:**

To foster innovation and optimization in engineering product design, it is crucial for engineering professionals to effectively integrate knowledge and make informed decisions within interdisciplinary collaborative environments. Understanding the factors that influence group decision-making performance can enhance communication and knowledge integration among experts from diverse disciplinary backgrounds. By analyzing decision-makers’ attention allocation and information processing at the cognitive level, the innovation and practicality of solutions can be significantly improved. However, the complexity and multitude of factors affecting decision-making performance pose challenges, particularly due to the lack of quantitative research and unified metrics at both group and cognitive levels. This gap hinders the quality and efficiency of engineering group decisions.

**Methods:**

This study introduces an eye-tracking method to investigate interdisciplinary group decision-making in engineering design, leveraging group decision-making performance theory and eye-tracking technology. Experiments were conducted in the context of Chinese cruise ship cabin design. Using Partial Least Squares Structural Equation Modeling (PLS-SEM), a quantitative model was developed to assess the impact of visual attention on group decision performance.

**Results:**

The results demonstrate that group average gaze duration and group average number of gazes directly influence group decision-maker satisfaction and decision acceptability. Furthermore, these factors indirectly affect interdisciplinary group decision-making performance by impacting group decision quality.

**Discussion:**

The findings provide a foundation for developing effective interdisciplinary group decision support systems, enhancing cognitive performance, and offering new methodological insights for future engineering design decisions. This research contributes to bridging the gap in quantitative assessment of group decision-making performance, paving the way for improved decision quality and efficiency in engineering contexts.

## 1 Introduction

In the era of the knowledge economy, rapid technological development and innovation are the key forces driving social progress. Engineering product design, as an essential area of technological innovation, is greatly influenced by the multidisciplinary group decision-making process, which plays a decisive role in enhancing the innovation and practicality of products. Interdisciplinary group decision-making complexity arises from multiple levels: Firstly, teams must rapidly integrate knowledge and perspectives from multiple disciplines. Secondly, the process includes decision problem cognition, decision problem analysis, decision problem solving, and plan integration and selection (Feng and Chen, 2022), demanding coordination and integration at each stage. Lastly, many factors affect these decisions, such as cognitive differences, communication efficiency, processing capabilities, professional backgrounds, organizational culture, and environmental changes. These factors interact to shape the complexity and dynamics of group decision-making. Studying these factors reveals how subjective and objective elements affect outcomes, guiding measures to optimize group decision-making.

However, both academia and industry currently have a relatively superficial understanding of this group decision-making process. Existing research primarily focuses on qualitative studies from the perspectives of psychology, education, and management, and has not yet delved into quantitative analyses of the underlying mechanisms and influencing factors at the cognitive level of individual engineers (Parayitam et al., 2017). A significant gap exists in current theoretical research, which has resulted in a lack of unified and objective standards as well as calculation methods for evaluating group decision-making performance during the practical application of engineering design decisions. This deficiency leads to subjectivity and inconsistency in the evaluation of group decision outcomes. The primary reason lies in the limitations of traditional behavioral experiments, which are unable to effectively investigate the cognitive processes of individual engineers. Consequently, it becomes challenging to accurately capture cognitive dynamics, quantify cognitive states, or integrate professional knowledge with group consensus. As a result, gaining an in-depth understanding of the decision-making process remains elusive. Therefore, innovative experimental methods and research tools are urgently required to address these challenges and advance the field. The advancement of eye-tracking technology has equipped researchers with a tool for real-time measurement of cognitive activities. By recording eye movements, eye-tracking technology can accurately reflect an individual’s visual focus, thereby revealing their cognitive processing. This technology has been widely applied in fields such as cognitive psychology, human-computer interaction, and market research, but its application in interdisciplinary group decision-making research in engineering product design is still in its infancy (Zheng et al., 2024). This paper designs an eye-tracking experimental research method for the interdisciplinary group decision-making process in engineering product design and constructs a model of factors affecting group decision-making performance using partial least squares analysis and structural equation modeling. It identifies the attention allocation indicators that have the greatest impact on group decision-making performance, quantitatively analyzes decision-makers’ visual attention patterns at the cognitive level of engineering technical foci, reveals the relationship between interdisciplinary technical focus attention mechanisms and decision-making performance, and fills the gap in current research at the cognitive level. By taking the design of Chinese cruise ship cabin rooms as a case study, this paper analyzes how decision-makers’ visual attention allocation patterns affect the quality and efficiency of group decisions when facing interdisciplinary technical focus and proposes measures and recommendations to enhance interdisciplinary group decision-making performance from the perspective of visual cognition.

## 2 Literature review

### 2.1 Research on group decision-making performance measurement

*Group decision-making performance* is a multidimensional outcome variable that involves assessments at multiple levels. Existing studies commonly use the Analytic Hierarchy Process (AHP) to break down group decision-making performance into several components, including, but not limited to, decision quality, efficiency, participant satisfaction, and emotional acceptance (Feng and Chen, 2022). The relative importance of these factors is determined through pairwise comparisons or expert evaluations, which are then used to calculate the weight of each decision factor (Jaafar, 2017).

*Decision quality* is a key indicator for evaluating the contribution of a decision to achieving organizational goals, and it is assessed and measured using subjective and objective analysis methods (Ge et al., 2021). The subjective analysis method primarily relies on survey scales to collect team members’ subjective perceptions of decision quality, which are usually tailored according to the research question. Jana et al. (2017) proposed using interval values in the survey scales to represent the subjective uncertainty of decision quality. Dong (2011) designed a decision quality scale from a business perspective, focusing on how the group’s daily performance and future growth potential affect decision quality. The objective analysis method is more commonly applied in behavioral simulation studies. Researchers typically design cases with a clear optimal outcome, assessing decision quality by comparing the gap between participants’ choices and this optimal result. These approaches are crucial for developing a more systematic understanding of group decision-making processes and their outcomes (Arvai and Froschauer, 2010). In addition, the studies by Mesmer-Magnus and Dechurch (2009) and Reimer et al. (2010) explored the impact of information sharing on decision quality through experimental methods, demonstrating the effectiveness of objective analysis methods.

*Decision efficiency* refers to the efficiency with which various factors—such as decision-makers, decision systems, and the operating environment—coordinate and cooperate according to certain rules to complete a specific task in a particular application context (Ming and Huchang, 2024). Zhang et al. (2018) proposed, from the perspective of group decision-making consensus, that consensus efficiency is derived through five comparative criteria: the adjusted number of decision-makers, the adjusted number of alternatives, the adjusted number of preference values, the distance between original preference information and adjusted preference information (adjustment cost), and the number of negotiation rounds required to reach consensus.

*Decision participant satisfaction* refers to the extent to which team members experience positive and enjoyable feelings during the decision-making process, making them willing to work in the same team again. Researchers have consistently emphasized the necessity of focusing on lower-level analyses to consider the satisfaction of individual team members. The studies by Crijns et al. (2023) and Torre-Ruiz et al. (2014) focused on the formation of individual satisfaction during team decision-making, with results indicating that communication within the team and the decision-making process significantly impact individual satisfaction. Cai et al. (2024) determined the decision maker’s satisfaction with the attributes in different alternatives by organising large groups of people to provide opinions in the form of linguistic variables and ranking the linguistic opinions by optimising the model weights.

*Decision acceptability* refers to the extent to which the decision outcome is accepted by both internal and external stakeholders. The assessment of decision acceptability often employs subjective analysis, which involves calculating acceptance scores or external feedback. Erickson et al. (2014) and Mee and Young (2018) developed and modified the Team Function Scale (TFS), refining its content through in-depth interviews, content validity assessments, and internal consistency reliability tests to more accurately capture and evaluate overall team functioning during the decision-making process. Building on this foundation, Sun and Xin (2017) proposed the “Process-Outcome Conceptual Model” for group decision evaluation, assessing real group decisions from four dimensions: information processing, interpersonal interaction, objective task outcomes, and subjective emotional outcomes.

Current research has made progress in the multidimensional analysis of group decision-making performance; however, there is a lack of clear definitions and measurement methods for the quantitative assessment of each performance dimension, and a unified standard to weigh the proportion of subjective and objective survey results is missing (Parayitam et al., 2017). Moreover, there is a significant deficiency in measuring interdisciplinary group decision consensus in existing studies. The formation of consensus facilitates effective information integration and knowledge sharing, reduces conflicts and misunderstandings in the decision-making process, and is crucial for ensuring decision efficiency and quality (Chae and Lee, 2013). It is also key to fostering effective communication and collaboration among decision-makers from different professional backgrounds. The absence of related research prevents the revelation of how disciplinary knowledge differences affect the degree of consensus through behavioral analysis, necessitating cognitive research tools for further exploration.

### 2.2 Application of attention allocation indicators in group decision-making

The application of eye-tracking technology provides a new perspective for interdisciplinary group decision-making research. By quantitatively measuring various attention allocation indicators, it has become an effective means of studying participants’ cognitive activities and visual focus. In the process of visual information processing, fixation behavior manifests as a relative state of stillness of the eyes, reflecting the subjects’ reception and processing of information. Compared to other cognitive measurement tools, the non-invasive, real-time, and high spatial resolution characteristics of eye-tracking technology make it an ideal tool for studying visual attention allocation (Li, 2018). Research by Chae and Lee (2013) demonstrated that gaze tracking is an efficient method for collecting individual cognitive states during decision-making. Via et al. (2018) explored the impact of responsibility types and causal chains on decision quality using eye-tracking technology, noting that the primary indicators obtained from eye-tracking devices are fixation duration and frequency. By measuring attention allocation indicators, researchers can reveal decision-making strategies and gain insights into underlying cognitive mechanisms (Zheng et al., 2024).

#### 2.2.1 Group average fixation duration ratio

The group average fixation duration ratio, also known as “fixation point duration,” describes the length of time an individual visually fixates on a specific Area of Interest (AOI) (Diao et al., 2017). In the context of group decision-making in engineering, AOIs are multidisciplinary technical focal points, such as key semantics in engineering data charts or design proposals. A longer fixation time implies that the brain is engaging in deeper information processing for that area, which may indicate that the area is more attractive to the individual, or that the individual finds it difficult to extract information from the presented content (Amit et al., 2016). Eye-tracking devices can capture the fixation durations of all decision team members on each AOI.

#### 2.2.2 Group average fixation count

Group average fixation count refers to the number of fixations within an AOI, which reflects an individual’s understanding and attention to the presented information (Laoura and Athanassios, 2023). In the context of group decision-making in engineering, the group average fixation count is also an indicator of the importance of an AOI (technical focus). Eye-tracking devices can capture the fixation counts of all decision team members on each AOI, and the average of these fixation counts for a given AOI represents the group average fixation count. Fixation duration and count have similarities in explaining information processing; however, existing studies have overlooked the dynamics of group interactions and collective attention patterns, thus failing to explain how group average fixation duration ratio and group average fixation count jointly affect the group decision-making process (Matthew and Roger, 2023).

#### 2.2.3 Group fixation heatmap overlay distribution

Fixation heatmaps visually illustrate the differences in team members’ attention to various AOIs (technical focal points) through variations in color intensity. Yan et al. (2018) pointed out in related studies that meaningful results can be obtained when these attention differences are linked to behavioral outcomes, such as learning or decision-making performance. In the context of group decision-making in engineering, eye-tracking devices can capture the fixation heatmaps of all team members during the decision-making process.

#### 2.2.4 Group fixation trajectory consistency

According to eye movement trajectory analysis theory, eye movement is a dynamic process guided by internal cognitive models, mapping the sequence of visual stimuli processing in the brain (Huang et al., 2020). Currently, researchers use various quantitative analysis methods to study eye movement trajectories, including string-based analysis, probabilistic models, and geometric vector methods. The main steps include preprocessing fixation data, defining and coding areas of interest, forming eye movement trajectory sequences, and calculating similarity scores to compare the similarity of two eye movement trajectories, where the similarity score reflects similarity or difference in cognitive processes (Tjon et al., 2023; Zachary et al., 2022). Although fixation trajectory consistency provides a new perspective for understanding cognitive synchronization among team members, existing literature still lacks in-depth exploration of how group fixation behavior interacts with decision-making performance.

While the attention allocation indicators offer a new perspective for understanding cognitive synchronization among team members, there is a dearth of in-depth exploration in the literature regarding how group gaze behaviors interact with decision-making performance, and the quantitative relationships and mechanisms of influence between these indicators and decision performance remain unclear (Zachary et al., 2022). Researchers have begun to explore how these indicators reflect individual decision strategies and cognitive mechanisms, but there is a research gap concerning their collective impact on group decision-making processes and the specific pathways through which they affect group decision performance. This is partly due to technological and cost limitations of eye-tracking equipment, which complicate data collection and analysis at the group level. Additionally, methods for analyzing group dynamics and collective attention patterns are immature, necessitating exploratory work at the group level to understand the mechanisms of eye-tracking indicators in group decision-making processes.

### 2.3 Summary

Existing research on interdisciplinary group decision-making in engineering product design, group decision-making performance theories have proposed relevant measurement dimensions. However, these have primarily focused on the behavioral performance of decision-makers, neglecting the establishment and quantitative analysis of cognitive structures at individual and group levels, which are essential for explaining the mechanisms by which various factors influence group decisions. Measurement methods often rely on subjective scales to assess decision-makers’ behavioral performance, whereas eye-tracking technology offers a new perspective and method for quantifying decision-makers’ cognitive behaviors. Despite this, previous studies have not sufficiently explained or quantitatively analyzed how eye-tracking indicators relate to multiple dimensions of group decision-making performance, including quality, efficiency, participant satisfaction, and acceptability, and how these indicators impact the decision-making process and outcomes. Building on a systematic review of relevant literature, this paper employs eye-tracking experimental design and attention allocation indicators to explore the attention allocation mechanisms of decision-makers regarding technical foci and their impact on group decision-making performance. A quantitative model is constructed using Partial Least Squares Structural Equation Modeling (PLS-SEM) to reveal the mechanisms by which visual attention allocation affects group decision-making performance. This study delves into the attention allocation mechanisms for technical foci and the factors influencing group decision-making performance in interdisciplinary contexts, filling gaps in previous research and providing new theoretical support and empirical evidence for future research and practice.

## 3 Experimental process

**Figure 1:**
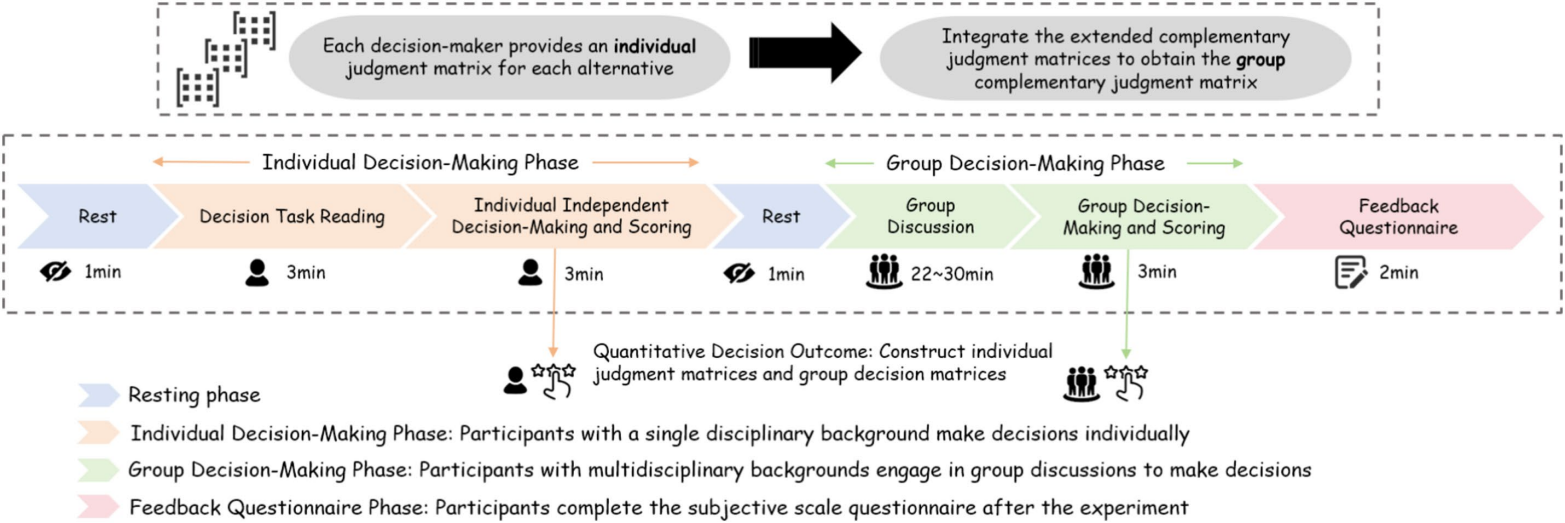
Flowchart of eye-tracking experiment for interdisciplinary decision-making in engineering product Design.

Building on the literature review, this study aims to conduct empirical research on group-level eye-tracking cognitive measurement experiments to explore the specific link between group-level attention allocation and group decision-making performance. The experiment consists of two phases: the individual decision-making phase with a single disciplinary background and the group decision-making phase with a multidisciplinary background. Upon arriving at the laboratory, each group of participants first signed an informed consent form and then completed an anonymous basic information questionnaire. The basic information questionnaire contains 10 questions aimed at collecting demographic data such as participants’ age and gender, as well as their academic background and engineering project experience. Following this, participants proceeded to the eye-tracking signal measurement stage. The experimental process flowchart is shown in Figure 1. It should be noted that, due to the individual decision-making stage and group decision-making stage for the same subjects in the same decision-making environment for the same decision-making content, and the two decision-making stages are separated by a short time, does not have the potential learning effect.

**Figure 2:**
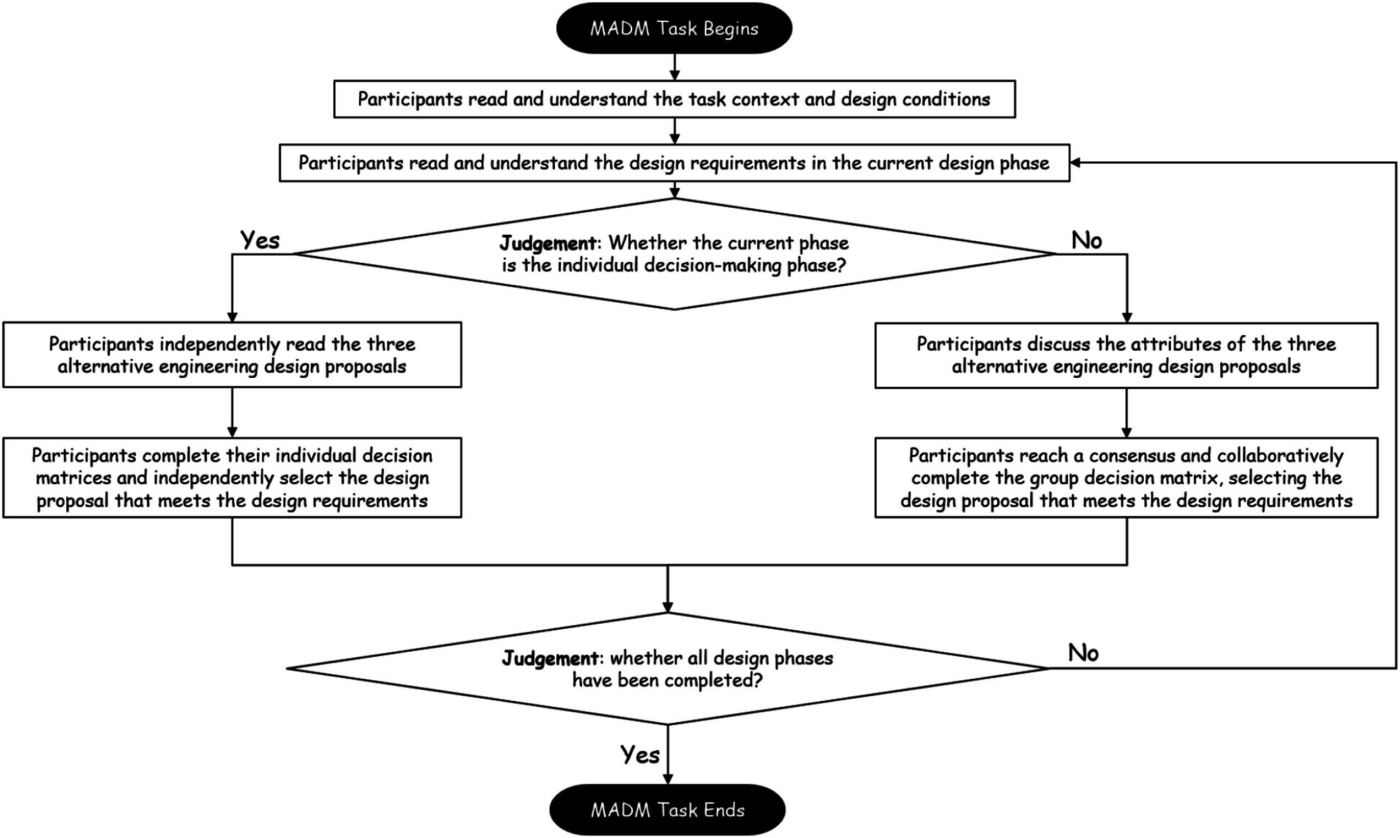
Multidisciplinary group discussion MADM task paradigm flowchart.

This experiment employs a multi-attribute decision-making task (MADM), considering the ranking or selection of limited alternatives based on multiple attributes. For interdisciplinary decision-making groups, this study introduces a group discussion phase based on the MADM task, proposing an interdisciplinary group discussion MADM task paradigm. Participants are required to use their disciplinary knowledge to discuss various attributes and selection criteria of the alternatives with participants from other disciplines. According to the Concept-Knowledge theory (C-K theory), interdisciplinary technical foci within the decision task can construct the cognitive structures in participants’ minds. The think-aloud protocol is employed to effectively capture fragments of participants’ thoughts, and thus participants are required to verbally express themselves using their disciplinary technical foci (disciplinary terms) during the group discussion phase (Li, 2018). Upon completion of all design phases, the interdisciplinary group discussion MADM task will conclude (showed in Figure 2).

The interdisciplinary group discussion MADM task proposed in this study mainly collects two types of data: first, the alternative evaluation matrix filled out by participants during the MADM task, with numerical values reflecting the participants’ decision outcomes. Second, participants’ attention allocation results regarding the technical focus during the MADM task, which, together with the decision outcomes, form a “cognitive level-decision performance” structure. This structure will serve as an explicit representation of the participants’ cognitive structure toward the design task objects in the interdisciplinary group discussion MADM task, revealing the influence direction of interdisciplinary technical foci on decision cognition and decision performance.

## 4 Case selection, participant recruitment, and system development

### 4.1 Selection of experimental paradigm

The design of cruise ship cabins, as a typical scenario in interdisciplinary engineering product design, is a classic multi-attribute decision-making (MADM) task, completed through collaborative decision-making by scholars from structural engineering, design engineering, and environmental engineering. In the multidisciplinary design of cruise ship cabins, participants with different disciplinary backgrounds emphasize different attributes of the design object. According to the survey results, participants with a structural engineering background tend to focus more on the functionality and practicality of the design, such as the arrangement of storage space and the layout of lighting and windows. Participants with an environmental engineering background are more inclined to consider the comfort of passengers, such as temperature and air quality control and noise isolation. Participants with an aesthetics background place greater emphasis on aesthetics and brand image. For different types of cruise ships, such as family-oriented, luxury, or themed types, cabin design should match the specific type and enhance its aesthetics by selecting appropriate colors, materials, and decorations. This type of task, which involves making an optimal choice by considering multiple factors, is a multi-attribute decision-making (MADM) task.

In terms of simulating multidisciplinary knowledge integration, the experiment involves a simulated real-world cruise ship cabin design task, encouraging participants with different disciplinary backgrounds to integrate their specialized knowledge to collaboratively solve design problems. This simulation mirrors multidisciplinary collaboration in engineering practice, providing a realistic research environment. The knowledge used in experimental design comes from the textbooks “*Ship Aesthetics and Cabin Design*” (Yang and Wang, 2021) and “*Research and Design of Ship Cabin Environmental Engineering*” (Yang, 2020). The decision-making materials were selected based on the following considerations for typicality:

1. The integrity of the knowledge system: The textbook comprehensively introduces cabin design, covering five major aspects: cabin internal structure and layout, color environment, lighting environment, cabin insulation design (air environment), and noise environment, ensuring the comprehensiveness and systematic nature of the experimental materials at the knowledge level.
2. The integration of multidisciplinary knowledge: The book integrates theories and practices from aesthetics, ergonomics, environmental science, and other disciplines, which is highly aligned with the multidisciplinary group decision-making theme of this study.
3. The richness of practical cases: Through specific case analyses, it illustrates the application of ship aesthetics in actual ship modeling and cabin design, providing rich context simulations and discussion materials for the experiment.
4. The advancement of design methods: The textbooks were published in 2020 and 2021, and the design concepts and methods reflect the latest developments in ship design, helping to stimulate in-depth discussions among participants.

### 4.2 Selection of engineering concept displays

Regarding the selection of technical focus, the standard experimental scheme provided by “*Research and Design of Ship Cabin Environmental Engineering*” (Yang, 2020) serves as the input data. After preprocessing steps including text cleaning, text classification (categorizing texts based on disciplinary knowledge from the standard scheme), tokenization, and stopword filtering, the processed text data is input into a named entity recognition algorithm to extract technical focus entities. To determine the disciplinary classification of these technical focus terms, a corresponding interdisciplinary knowledge network is constructed with technical focus entities as nodes and the frequency of their occurrence in the text data as edges. Through this approach, nodes from the same discipline naturally cluster, allowing classification based on disciplinary affiliation, which is recorded in Table 1. The single-discipline technical focus categories include Structural Engineering (SE), Aesthetic Design (AD), and Environmental Engineering (EE). Furthermore, by employing complex network analysis to calculate the centrality of nodes, entities with higher complexity, referred to as High Centrality Network terms (HCN), can be identified. If these entities appear in multiple disciplinary clusters, they are classified as interdisciplinary technical focus and recorded in Table 1. It is important to note that the interdisciplinary technical focus network serves solely as a classification tool, and only the extraction and classification results of technical focus are presented here.

**Table 1:** Engineering technical focus and their respective disciplines in multidisciplinary alternatives.

HCN	Technical focus (HCN)	SE	Technical focus (SE)	AD	Technical focus (AD)	EE	Technical focus (EE)
HCN_1	Cabin internal structure and layout	SE_1	Semi-partition form	AD_1	Light coffee-colored carpet	EE_1	Rectangular ceiling light
HCN_2	Color and light environment	SE_2	Lower part of partition	AD_2	Dark blue carpet	EE_2	Molecular ball-shaped colored light
HCN_3	Cabin insulation design and noise environment	SE_3	Steel material	AD_3	Light gray sofa	EE_3	Natural and soft yellow light
HCN_4	Double-layer marine acrylic glass with blinds	SE_4	Half-height wing wall (rigid partition)	AD_4	Dark blue sofa	EE_4	Bright white light
HCN_5	Wool	SE_5	Wood material	AD_5	Single-layer steel bulkhead combined with magnesium oxide fireproof board	EE_5	Color temperature, illuminance
HCN_6	Synthetic fiber	SE_6	Storage rooms, lavatories arranged around bedroom	AD_6	Single-layer steel bulkhead combined with rock wool board	EE_6	Insulation
HCN_7	Insulation material	SE_7	Storage rooms, lavatories arranged in noise isolation area	AD_7	Single-layer steel bulkhead combined with glass		

### 4.3 Selection of decision-making materials

According to the definition of the MADM task for cruise ship cabin design, the experimental case must meet the following criteria:

1. The case must involve a problem of selecting or comparing multiple alternatives;
2. Each alternative must contain multiple different attributes for decision-makers to compare;
3. To ensure the reasonableness and accuracy of group scoring, the number of attributes for each alternative must be consistent with the number of decision-makers.

Based on these requirements, three design alternatives were developed using the “VIP Cabin Design Example of a Ro-Ro Passenger Ship” provided by “Research and Design of Ship Cabin Environmental Engineering” (Yang, 2020; as shown in Table 2), for evaluation and selection by a multidisciplinary group decision-making team. This case focuses on cabin design and includes five design aspects: spatial planning and layout, color environment, light environment, air environment, and noise environment. Considering that the MADM task for cruise ship cabin design only involves knowledge from three disciplinary fields—structural engineering, aesthetics, and environmental science—the attributes of the alternatives for the multidisciplinary group decision-making in this experiment were modified to three: “cabin internal structure and layout,” “color and light environment,” and “cabin insulation design and noise environment.”

**Table 2:** Proposed design alternatives for ship cabins.

Decision content	Option A	Option B	Option C
Cabin internal structure and layout	The bedroom and office area adopt a semi-partition format. The lower part of the partition uses steel materials. The upper part is a double-layer marine acrylic glass with blinds.	The bedroom and office area adopt a semi-partition format. The partition is in the form of a marine half-height wing wall (rigid partition). The upper part is a double-layer marine acrylic glass with blinds.	The bedroom and office area adopt a semi-partition format. The lower part of the partition uses wood materials consistent with the bulkhead. The upper part is a double-layer marine acrylic glass with blinds.
Color and light environment	The office area has a light coffee-colored glass wool carpet and a light gray sofa. The office area is equipped with a rectangular ceiling light (40 W). The bedroom bedside lamp uses soft yellow natural light (color temperature 2,800 K, illuminance 100 lx).	The office area has a light coffee-colored pure wool carpet and a light gray sofa. The office area is equipped with a rectangular ceiling light (40 W). The bedroom bedside lamp uses soft yellow natural light (color temperature 2,800 K, illuminance 100 lx).	The office area has a dark blue glass wool carpet and a dark blue sofa. The office area is equipped with a molecular ball-shaped colored light (15 W). The bedroom bedside lamp uses bright white light (color temperature 3,500 K, illuminance 200 lx).
Cabin insulation design and noise environment	The cabin bulkhead uses a combination of single-layer steel bulkhead and magnesium oxide fireproof board as insulation material. Insulation thickness is 50 mm. Storage rooms, lavatories, etc., are arranged around the bedroom.	The cabin bulkhead uses a combination of single-layer steel bulkhead and rock wool board as insulation material. Insulation thickness is 25 mm. Storage rooms, lavatories, etc., are arranged around the cabin ceiling as noise isolation areas.	The cabin bulkhead uses a combination of single-layer steel bulkhead and glass as insulation material. Insulation thickness is 50 mm. Storage rooms, lavatories, etc., are arranged around the cabin ceiling as noise isolation areas.

In terms of decision consensus and conflict design, the MADM task for cruise ship cabin design requires a multidisciplinary team with backgrounds in structural engineering, aesthetics, and environmental science to design the ship cabin. The decision-making task involves the team of three evaluating the feasibility of the three alternative design proposals shown in Table 2 (denoted as Options A, B, and C) and providing a final team decision. Since the number of alternatives exceeds the number of final selections, it meets the requirement for reaching group decision consensus, defined as a “state of mutual agreement among group members.” Table 2 compares three design options (Option A, B, and C) for ship cabin designs, focusing on differences in internal structure/layout, color/light environment, and thermal insulation/noise control. Option A employs steel partitioning at the lower section with double-layer acrylic glass at the upper section, light-colored carpets, and soft yellow lighting. Option B uses semi-high rigid partitions (marine half-height wing walls) with the same flooring materials and lighting design as Option A. In contrast, Option C adopts wooden or dark-blue-themed partitions, dark-colored carpets, and high-brightness white lighting. Regarding thermal insulation and noise control, Option A utilizes magnesium oxide fireproof boards, Option B employs rock wool panels, while Option C uses glass for insulation purposes. Additionally, the arrangement of storage spaces in Option C differs from the other two designs.

To ensure that decision-makers from different disciplines experience cognitive conflict and reach consensus based on the options, all three design proposals are designed with multidisciplinary content in the three attribute dimensions. For example, in the “cabin internal structure and layout” attribute dimension, in terms of choice conflict, “partition material must be the same as the bulkhead” is knowledge belonging to structural engineering, leading structural engineering decision-makers to prefer Option B or Option C. In terms of cognitive conflict, “half-height wing wall” is a specialized technical focus of structural engineering, which may create cognitive barriers for decision-makers in aesthetics and environmental engineering, leading to cognitive conflict. In this dimension, Option C is the closest to the standard proposal (Table 3).

**Table 3:** Recruitment criteria for experimental participants.

Criteria	Ship structure/Architecture	Aesthetics/Industrial design	Environmental engineering
Academic background	Possessing or soon to obtain a bachelor’s, master’s, or doctoral degree in Engineering Mechanics/Ship and Ocean Engineering/Architecture, having systematically studied courses such as Theoretical Mechanics, Material Mechanics, Structural Mechanics (or Structural Dynamics, Structural Design Principles).	Possessing or soon to obtain a bachelor’s, master’s, or doctoral degree in Industrial Design/Environmental Design/Art Design, having systematically studied courses such as Interior Environment Design, Design Drawing and Perspective.	Possessing or soon to obtain a bachelor’s, master’s, or doctoral degree in Environmental Science and Engineering/Environmental Science/Environmental Engineering, having systematically studied courses such as Environmental Engineering Principles, Environmental Monitoring (Technology).
Project experience	Internship and project experience in Ship Structure/Industrial Design/Environmental Engineering for at least 3 months.		
Vision	Normal unaided or corrected vision in both eyes, no strabismus, and no history of corrective eye surgery.		
Other	① No history of mental illness; ② Adequate reading comprehension and verbal communication skills for survey questionnaires and technical documents; ③ Ability to perform basic computer operations according to monitor prompts.		

### 4.4 Recruitment criteria for experimental participants

Considering the ability to complete engineering design tasks and the corresponding knowledge reserve of engineering experience, the recruited participants must meet the following criteria:

The experiment recruited 24 groups of participants from the faculty and students at the School of Mechanical Engineering at Shanghai Jiao Tong University and from a shipbuilding company. A total of 24 qualified participant groups (72 individuals) were recruited. The participants had an average age of 25.17 years, with a standard deviation of 4.61 years; among them, 47 were male and 25 were female. There were 40 undergraduate students, 24 masters, and 8 doctors. All 72 participants had either participated in engineering internships or were currently employed within the past year.

### 4.5 Software and hardware for the experiment

**Figure 3:**
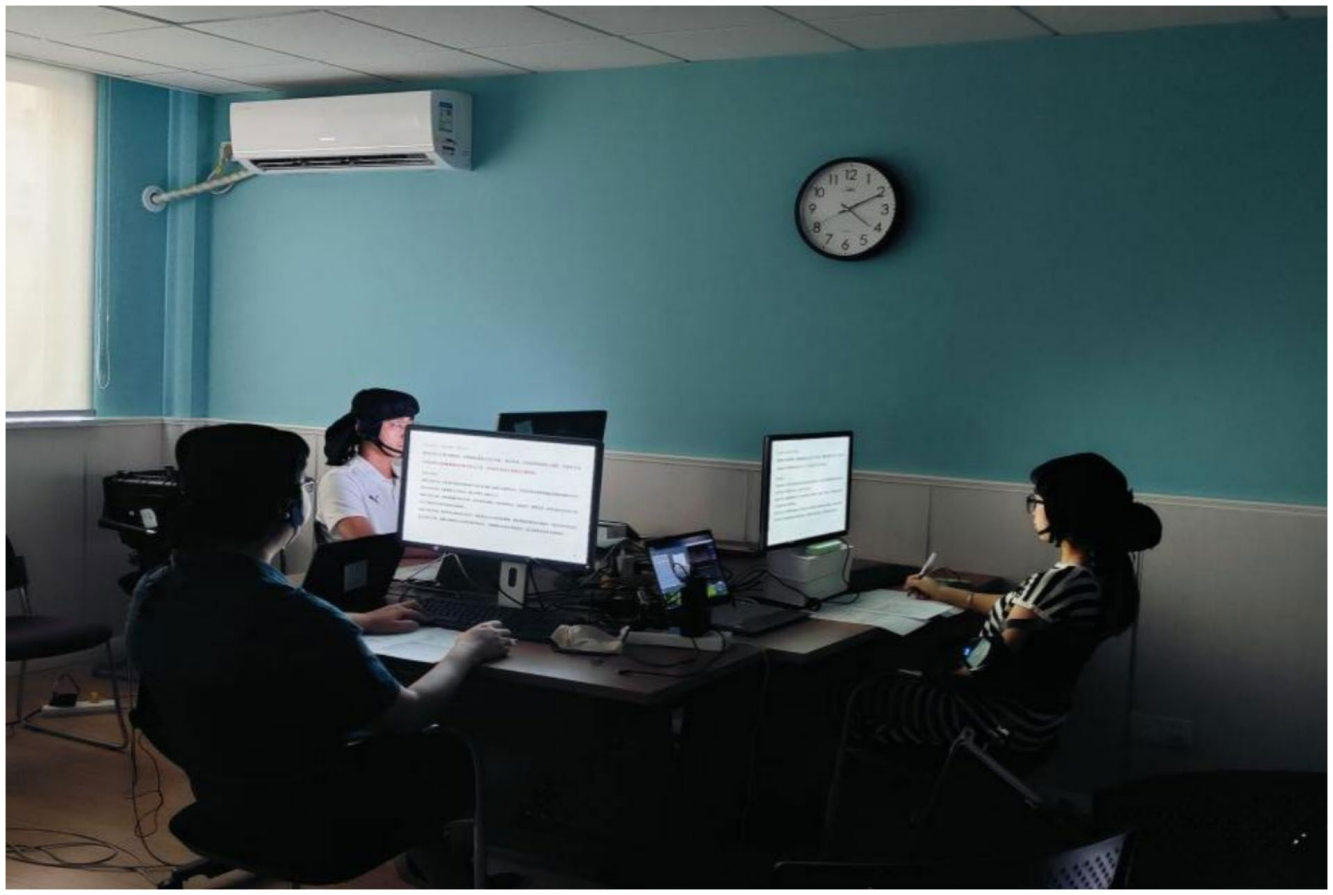
Participant experimental setup and equipment layout diagram.

The experiment was conducted in an independent, quiet laboratory environment. The experimental system ran on a computer with 4GB of memory and a Core i5 processor, and the experimental content was displayed to participants via a 1920 × 1,080 pixel, 60 Hz refresh rate monitor (display area width 507 mm, height 283 mm). Using the built-in experiment design functionality of Experiment Center software, the three alternative group decision design schemes were imported in the form of images. Participants only needed to press the spacebar to sequentially browse the full-screen presentation of the design scheme on the computer screen. Once the experiment started, participants followed the instructions from the experimental system and completed the corresponding operations using the keyboard. Eye movement data were recorded using an Eye-Logic eye tracker fixed below the screen, with a sampling frequency of 60 Hz. To collect verbal feedback from participants, the entire experiment process for all participants was recorded using screen recording software with audio capabilities. The experimental setup, including participant conditions and the geometric relationships of the equipment, is shown in Figure 3.

## 5 Experimental data acquisition and statistic results

### 5.1 Raw data collection

The experiment collected the following three types of raw data:

1. Basic participant information, such as gender, age, and self-reported experience, through a basic information questionnaire.
2. Decision-making processes and time consumption during both individual decision-making and group decision-making phases were recorded using EV screen recording while running the decision-making experiment program via Experiment Center software.
3. Eye movement fixation data for participants performing MADM tasks during both individual decision-making and group decision-making phases were collected using the Eye-Logic eye tracker and Experiment Center software’s data synchronization system.

### 5.2 Quantitative representation of research factors

#### 5.2.1 Group decision-making performance

Commonly used metrics for evaluating group decision-making performance include decision quality, decision efficiency, participant satisfaction, and emotional acceptability. In the engineering field, participants with different disciplinary backgrounds may have varying understandings and preferences for the same issue. This requires that, when evaluating group decision-making performance, not only should the overall outcome be considered, but the judgments of participants from different disciplinary backgrounds on each decision attribute must also be thoroughly understood to quantify the degree of consensus among participants with different backgrounds. Therefore, in calculating the multidisciplinary group decision-making performance in engineering, this study adds the key variable of consensus degree in addition to traditional metrics such as decision quality and efficiency, to more accurately assess and optimize the group decision-making process.

##### 5.2.1.1 Decision quality

In this experiment, the decision scheme design was adapted from a standard design example—the luxury cruise VIP cabin design example—providing a clear and quantifiable optimal outcome standard, which meets the conditions for using objective analysis. However, due to the nature of group decision-making, which requires all members to reach consensus, it cannot be guaranteed that every decision-maker will have the same level of recognition regarding the decision outcome’s quality. Therefore, this study uses an adapted decision quality measurement scale, combining subjective and objective analysis methods to provide a more comprehensive assessment of decision quality. The weights of the two methods are determined by data variance, as described in section 4.3.2.

Assuming there are p decision alternatives with q decision dimensions, for the k−th decision-maker (k=1,2,…,m), the decision results are recorded using a multi-attribute decision matrix. The individual decision matrix is $D^{\left(k\right)}={\left(d_{ij}^{\left(k\right)}\right)}_{p\times q}$. In the multidisciplinary group decision experiment, the participants’ differences lie only in their disciplinary backgrounds, so the weights of individual decision matrices within the same group are considered identical. Therefore, the group decision matrix can be denoted as (Equation 1):


(1) $$D_{ij}^{\left(G\right)}=\frac{1}{m}\sum_{k=1}^{m}d_{ij}^{\left(G\right)}$$

The group decision matrix for the standard scheme used in the experiment is denoted as $D_{standard}={\left(d_{i,j}\right)}_{p\times q}$. Let the decision-making quality be $y_{1}$, then $y_{1}$ can be calculated as (Equation 2):


(2) y1=Dij(G)·Dstandard‖Dij(G)‖‖Dstandard‖=∑i=1p∑j=1qdij(G)⋅di,j∑i=1p∑j=1q(dij(G))2∑i=1p∑j=1qdi,j12

##### 5.2.1.2 Consensus degree

In group decision-making, consensus is typically quantified based on the consistency of decision matrices. As mentioned previously, let there be m decision-makers in the experiment. For the k-th decision-maker (k=1,2,…,m), let the individual decision matrix be $D_{ij}^{\left(k\right)}$, and the group decision matrix be: $D_{ij}^{\left(G\right)}=\frac{1}{m}\sum_{k=1}^{m}d_{ij}^{\left(k\right)}$, The group decision matrix is then normalized to the maximum value to obtain the collective decision matrix $D_{ij}^{\left(G\right)}$. Let the consensus degree $CD\left\{e_{1},e_{2},\ldots ,e_{m}\right\}$ be denoted as $y_{2}$, then $y_{2}$ can be calculated as (Equation 3):


(3) $$y_{2}=1-\frac{1}{m\times n}\sum_{k=1}^{m}\sum_{i=1}^{n}\left|d_{ij}^{\left(k\right)}-d_{ij}^{\left(G\right)}\right|$$

##### 5.2.1.3 Decision efficiency

Based on the group decision consensus theory proposed by Zhang et al. (2018), decision efficiency is measured using indicators such as decision time, individual preference adjustment distance, group preference adjustment distance, and the number of discussion rounds required to reach consensus. In this experiment, the number of team members and the number of alternatives is fixed, while differences among experimental groups are in decision time, group preference adjustment distance, and number of discussion rounds. Therefore, three decision efficiency comparison standards are proposed:

1. *Decision time (T):* The time required for group discussion, measured in seconds.
2. *Group preference adjustment distance (D):* The distance between the decision results before and after group discussion. In group decision-making, Manhattan distance is widely used to measure the distance between preferences.
3. *Number of group decision discussion rounds (R):* In the experiment, participants take turns speaking in the order of “structural engineer—design engineer—environmental engineer.” Once all three have spoken, one discussion round is completed, and the round count is incremented by 1.

According to the definition of Equation 3 and the variable symbols, let the decision efficiency be $y_{3}$. Then $y_{3}$ can be calculated as (Equation 4):


(4) $$y_{3}=\frac{\frac{1}{m\times n}\sum_{k=1}^{m}\sum_{i=1}^{n}\left|d_{ij}^{\left(k\right)}-d_{ij}^{\left(G\right)}\right|}{T/R}$$

##### 5.2.1.4 Group decision participant satisfaction

Group decision participant satisfaction is primarily measured and analyzed using subjective scales. In this study, an adapted decision quality measurement scale was used to collect individual participants’ subjective ratings of their satisfaction with the experimental process. The scores were averaged and analyzed statistically, with the resulting group decision participant satisfaction denoted as the variable $y_{4}$.

##### 5.2.1.5 Group decision acceptability

Like group decision participant satisfaction, decision acceptability is usually assessed using subjective analysis. This involves calculating the acceptability scores based on team members’ subjective perceptions of decision acceptability or external feedback. The scores are then averaged and statistically analyzed, with the resulting group decision acceptability denoted as the variable $y_{5}$.

#### 5.2.2 Attention distribution on technical focuses

Considering the operability of the experiment and the accuracy of the results, this study selects participants who are native speakers of Chinese and specifically uses Chinese design materials as display materials for eye-tracking experiments. This choice is primarily based on several considerations: firstly, the widespread availability and ease of access to Chinese materials make it more feasible to construct experimental stimuli and control experimental conditions. Secondly, considering the readability of the materials, using the participants’ native language—Chinese—ensures that language comprehension barriers are minimized during the experiment, thereby more accurately capturing the participants’ visual attention allocation and cognitive processing.

**Figure 4:**
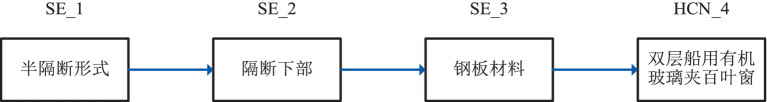
Eye-tracking trajectories and corresponding AOIs for cabin design tasks (partial).

In terms of metric acquisition, the Eye-logic eye tracker was utilized to collect data on participants’ fixation duration, fixation position coordinates, and fixation count. These data were automatically processed and aggregated using BeGaze software, enabling direct extraction of Attention Allocation Indicators through the interface. The software calculated the Group average fixation duration ratio and Group average fixation count using arithmetic mean methods. Additionally, the Group fixation heatmap overlay distribution was generated through Boolean union operations. By vectorizing the coordinates of each AOI to form eye movement trajectories (Figure 4 shows some of the gaze trajectories for the ship cabin design task, noted as the gaze trajectory vectors $\left\{\vec{\text{SE}\_1},\vec{\text{SE}\_2},\vec{\text{SE}\_3},\vec{\text{HCN}\_4}\right\}$), the Group fixation trajectory consistency was determined by computing the cosine similarity of these trajectories.

In this study, to reflect the actual decision-making habits of engineers, we define the drawing area (DWG) as an area of interest (AOI). However, compared to textual materials, the drawing area focuses more on specific visual elements and has limitations in representing interdisciplinarity. Therefore, in studying interdisciplinary group decision-making processes, the size of the DWG AOI is intentionally set smaller to highlight technical foci presented in textual information. This design aims to ensure that the experimental materials can more comprehensively capture the allocation of visual attention in interdisciplinary group decision-making, providing more accurate data support for analyzing the attention allocation mechanisms of decision-makers toward technical foci and their impact on group decision-making performance.

Technical focus information encompasses areas of interest with high network centrality-specific terms (HCN), engineering terminology in the field of ship structure engineering (SE), aesthetics design (AD), and environmental engineering (EE). By statistically analyzing the eye-tracking data on the gaze time and gaze count on the aforementioned four AOIs and the DWG and calculating the ratio of gaze time and gaze count on each AOI, we quantify the participants’ visual attention allocation on various content in multi-attribute decision-making tasks.

### 5.3 Experimental statistical analysis of results

#### 5.3.1 Statistical analysis of visual attention distribution

**Figure 5:**
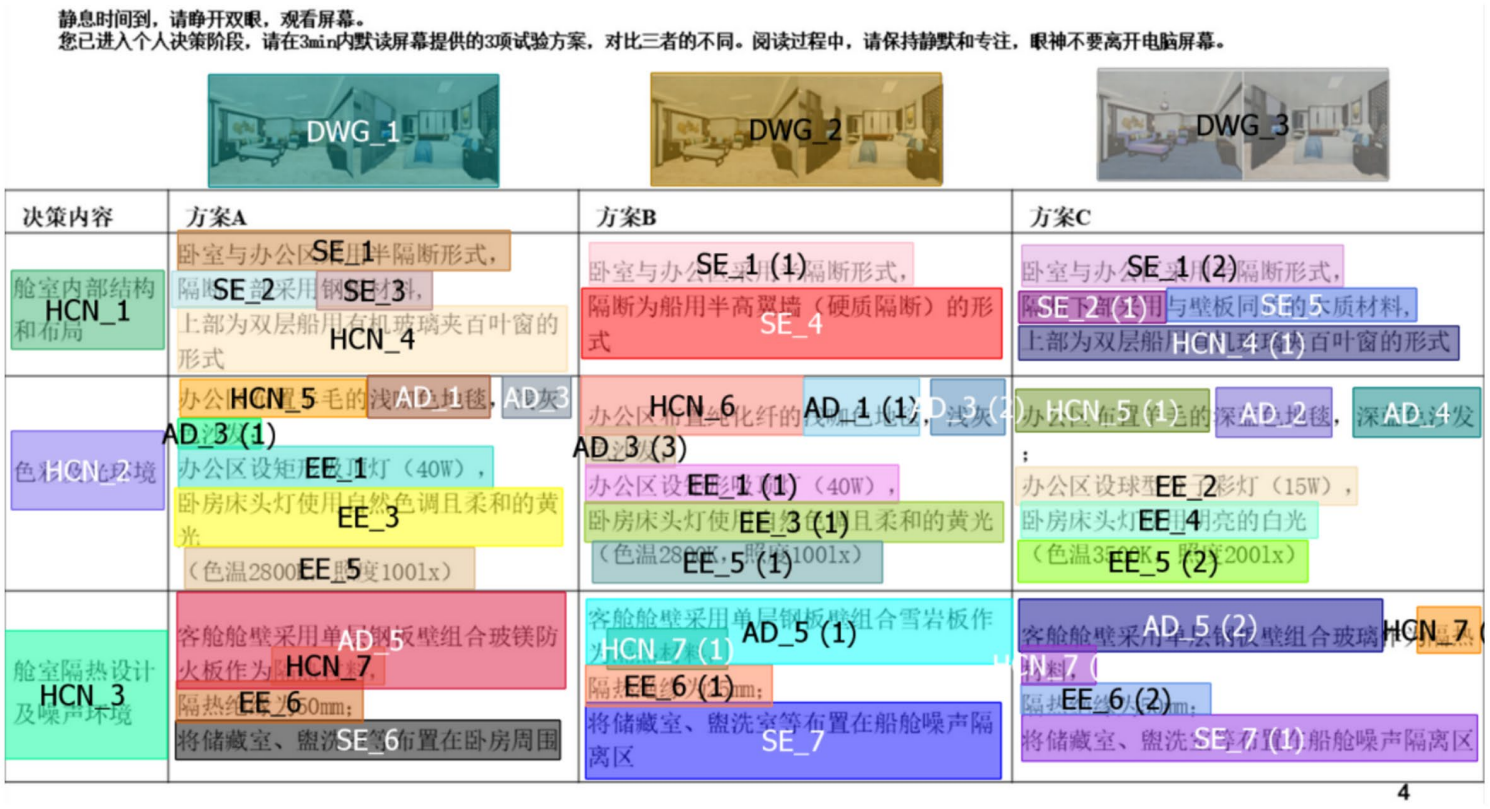
An example of areas of interest in the decision materials.

Figure 5 shows an example of the division of Areas of Interest (AOIs) in the ship cabin decision materials. High network centrality-specific terms (HCN), engineering terms from the ship structure field (SE), engineering terms from the design engineering field (AD), engineering terms from the environmental engineering field (EE), and blueprints (DWG) are marked with different colors, while unmarked areas are considered unrelated (others).

**Figure 6:**
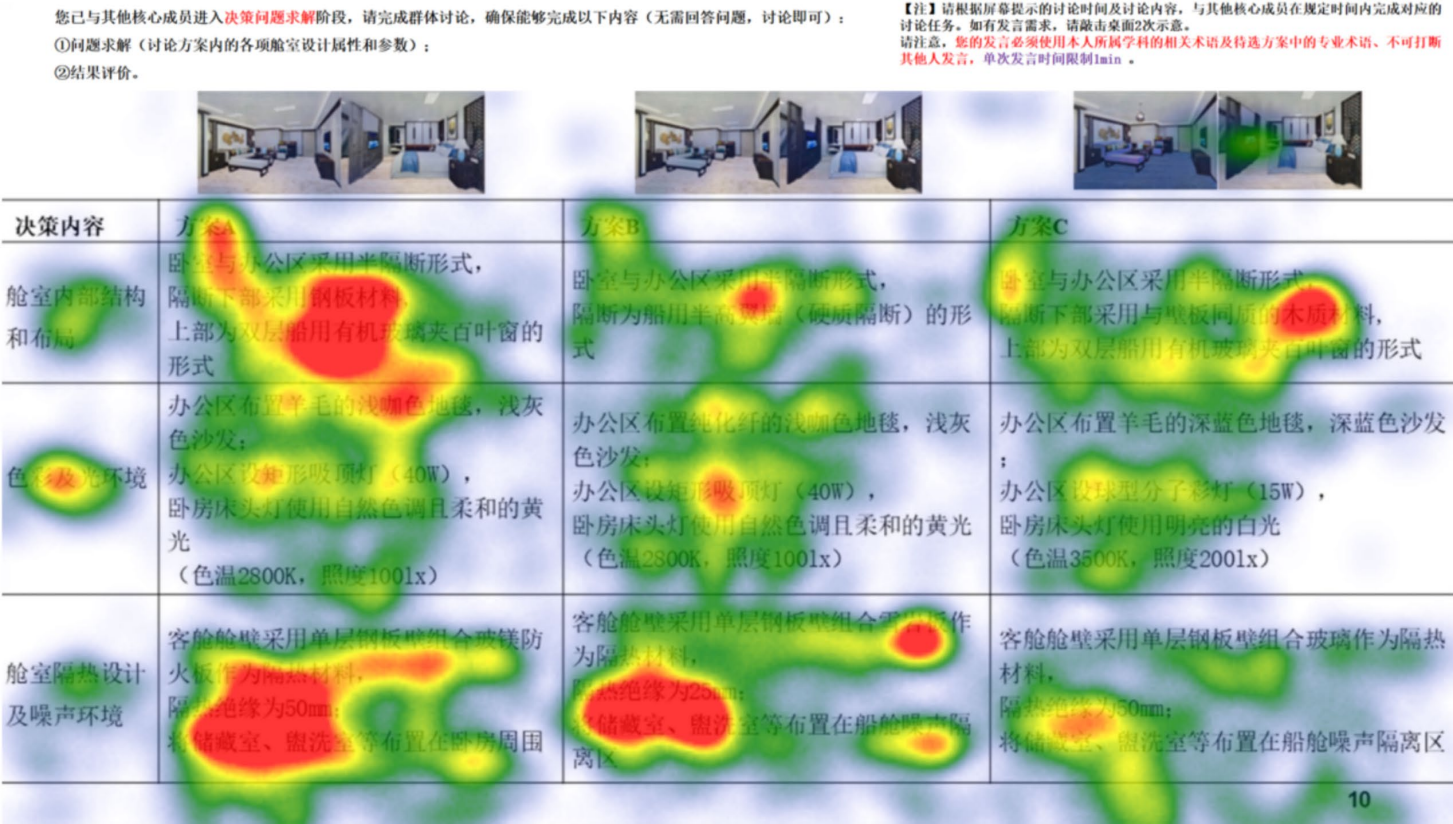
An example of fixation duration heatmap on learning materials.

The colored areas in Figure 6 represent the regions of primary focus for participants during the decision-making phase, with the red areas indicating regions where participants allocated more of their visual attention.

The visual attention distribution results for each Area of Interest (technical focus) in the decision materials are shown in Table 4. Participants’ visual attention was primarily distributed across the HCN, SE, AD, and EE areas, indicating that most of the 72 participants were able to focus on the information-dense parts of the decision materials. Additionally, the coefficient of variation for visual attention allocated to the DWG area was relatively high, suggesting significant variability in participants’ ability to discern image information, indicating considerable differences among the 72 participants.

**Table 4:** Visual attention distribution results for each area of interest in the decision materials.

Area of interest (AOI)	Fixation duration (time) ratio			Fixation count ratio		
	Mean	SD	CV	Mean	SD	CV
HCN	23.76%	5.27%	0.222	25.15%	4.36%	0.174
SE	30.54%	5.96%	0.195	29.66%	4.99%	0.168
EE	21.84%	3.89%	0.178	22.11%	3.24%	0.147
AD	17.41%	3.41%	0.196	18.30%	2.57%	0.140
DWG	6.47%	6.24%	0.966	4.80%	3.73%	0.778

#### 5.3.2 Group decision-making task performance statistics

In this experiment, the evaluation of group decision-making performance was characterized by a weighted combination of subjective and objective analyses. In the subjective scale survey, based on the collected questionnaire data, the mean scores for group decision quality, group decision consensus degree, group decision efficiency, outcome satisfaction, and acceptability were 4.345, 4.297, 4.525, 4.567, and 4.636, respectively. These mean values indicate that participants generally had a positive attitude toward the group decision-making process and outcomes. The variances for these metrics were 0.418, 0.399, 0.461, 0.470, and 0.426, respectively. These relatively small variances suggest a certain level of consistency among participants’ evaluations, although some individual differences were present.

In terms of objective analysis, group decision-making performance was evaluated by observing and recording participants’ behavior during the ship cabin design task. According to variable definitions, objective data for group decision outcome satisfaction and acceptability were not collected. Notably, the variance for group decision efficiency was relatively high, at 1.361. This is because, when calculating efficiency, participants’ behaviors were standardized to a scoring range of Feng and Chen (2022) and Jana et al. (2017). The occurrence of extreme values may have increased the variance, reflecting significant differences in group decision efficiency among participants, which could be related to individual work habits, decision-making styles, and team collaboration efficiency.

To more accurately evaluate group decision-making performance, a variance-weighted method was used to process the results of subjective and objective analyses, with the weight being inversely proportional to the variance. Notations can be checked in Table 5. For the independent variable $X_{i}$, let its objective analysis result be $X_{i,\text{obj}}$ and its subjective analysis result be $X_{i,\text{sub}}$. The weights are denoted as $\omega_{i,\text{obj}}$ and $\omega_{i,\text{sub}}$, respectively. Then ω_i_ and X_i_ can be calculated as (Equations 5,6):


(5) $$\left(\omega_{i,\text{obj}},\omega_{i,\text{sub}}\right)=\left(\frac{1}{\text{std}{\left(X_{i,\text{obj}}\right)}^{2}},\frac{1}{\text{std}{\left(X_{i,\text{sub}}\right)}^{2}}\right)$$

(6) $$X_{i}=\frac{\omega_{i,\text{obj}}\cdot X_{i,\text{obj}}+\omega_{i,\text{sub}}\cdot X_{i,\text{sub}}}{\omega_{i,\text{obj}}+\omega_{i,\text{sub}}}$$

**Table 5:** Notations of PLS-SEM.

Notation	Explanation
$X_{1}$	Group average gaze duration
$X_{2}$	Group average gaze times
$X_{3}$	(with DWG) Group gaze trajectory similarity
$X_{4}$	(without DWG) Similarity of group gaze trajectories
$Y_{1}$	Group decision-making Quality
$Y_{2}$	Group decision-making Consensus Degree
$Y_{3}$	Group decision-making efficiency
$Y_{4}$	Group decision-making Satisfaction
$Y_{5}$	Group decision-making Acceptability
$K_{i}\_\text{obj}$	The objective part of variable K(K=XorY)
$K_{i}\_\text{sub}$	The subjective part of variable K(K=XorY)

This method tends to assign higher weights to variables with lower variability, ensuring that the final evaluation results comprehensively reflect the actual situation of the group decision-making process and outcomes. The results after weighted analysis are shown in Table 6. By using this method, we can more accurately identify the key factors affecting group decision-making performance, providing a basis for subsequent interventions and improvements.

**Table 6:** Performance of group decision-making tasks for ship cabin design.

Performance dimension	Subjective analysis		Objective analysis		Weighted analysis	
	Mean	SD	Mean	SD	Mean	SD
Group decision quality	4.345	0.418	4.792	0.148	4.742	0.145
Group decision consensus degree	4.567	0.470	4.962	0.071	4.953	0.072
Group decision efficiency	4.297	0.399	3.708	**1.361**	4.165	0.414
Group decision outcome satisfaction	4.525	0.461	–	–	4.525	0.461
Group decision acceptability	4.636	0.426	–	–	4.636	0.426

Bold values indicates that the data set has a higher variance, proving that the data are more dispersed.

### 5.4 Structural equation model results

To explore the relationship between group decision-making performance (five dependent variables: group decision quality Y_1_, decision consensus degree Y_2_, group decision efficiency Y_3_, group decision outcome satisfaction Y_4_, and decision acceptability Y_5_) and the attention distribution on technical focus learning materials (four independent variables: group average fixation duration ratio on HCN, SE, AD, EE, and DWG areas X_1_, group average fixation count X_2_, group main trajectory similarity X_3_, and group fixation trajectory X_4_), as well as their causal relationships, a preliminary analysis was conducted to examine the correlations among all independent and dependent variables (Table 7).

**Table 7:** Correlation coefficient matrix for independent variables.

Variables	X_1_	X_2_	X_3_	X_4_
X_1_	1	0.857	−0.088	−0.334
X_2_	0.857	1	−0.04	−0.403
X_3_	−0.088	−0.04	1	0.173
X_4_	−0.334	−0.403	0.173	1

By constructing a correlation coefficient matrix for the independent variables and a correlation coefficient matrix for the dependent variables, it was found that there were certain correlations among the attention distribution on technical focus learning materials X_1_ to X_4_. In addition, there were also correlations among the five dimensions of group decision-making performance Y_1_ to Y_5_, as shown in Table 8. This indicates that these dimensions are interconnected to some extent. The presence of such correlations can affect the stability and interpretability of subsequent regression models.

**Table 8:** Correlation coefficient matrix for dependent variables.

Variables	Y_1_	Y_2_	Y_3_	Y_4_	Y_5_
Y_1_	1	−0.09	0.446	0.449	0.294
Y_2_	−0.09	1	0.086	0.021	0.063
Y_3_	0.446	0.086	1	0.825	0.743
Y_4_	0.449	0.021	0.825	1	0.876
Y_5_	0.294	0.063	0.743	0.876	1

To more accurately explore the relationships among the variables, Partial Least Squares (PLS) was used to construct a structural equation model. PLS reveals the predictive power of independent variables on dependent variables and is used for estimating model parameters. It is suitable for cases with small sample sizes, complex models, or high correlations among variables. The data preparation, parameter estimation, and model evaluation processes were all implemented using *SmartPLS 4.0* software.

Based on the correlations among the variables, the model assumes a direct linear relationship between the independent variables X_1_ to X_4_ and the dependent variables Y_1_ to Y_5_.

**Figure 7:**
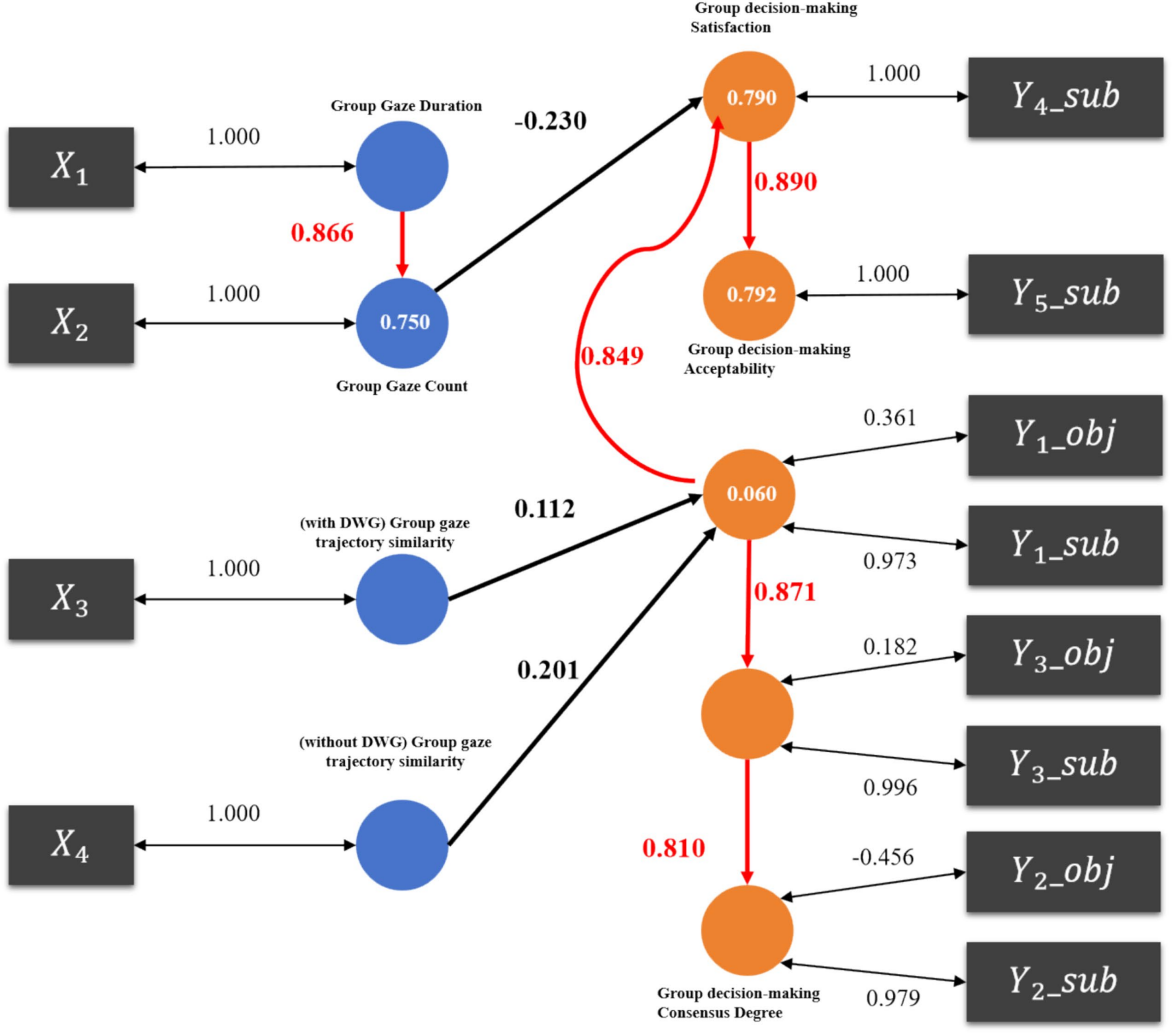
PLS model analysis results.

After conducting significance tests on the path coefficients of the model, the relationships among variables were adjusted by designating some variables as mediators (variables that act as intermediaries between independent and dependent variables) to enhance the model’s interpretability. The adjusted model hypotheses are illustrated in Figure 7. Specifically, X_2_ is designated as a mediator, connecting the independent variable X_1_ to the dependent variable Y_4_, and it also has a direct effect on Y_4_. And Y_1_ is designated as a mediator, linking the independent variables X_3_ and X_4_ to other dependent variables Y_3_ and Y_4_, and has direct effects on Y_3_ and Y_4_. The results of the PLS-SEM analysis are shown in Figure 6.

The model’s R^2^ scores (R-square) and adjusted R^2^ scores (R-square adjusted) are shown in Table 9. R^2^ is used to represent the proportion of variance in the dependent variable explained by the independent variables in a regression model. A higher R^2^ value indicates stronger explanatory power of the model. In practical applications, as the number of independent variables in the model increases, R^2^ can artificially increase even if the added variables do not substantially improve the model’s explanatory power. R^2^ adjusted accounts for the addition of unnecessary independent variables, providing a more balanced standard for comparing models. Therefore, researchers typically prioritize R^2^ adjusted when selecting and evaluating models.

**Table 9:** R^2^ scores of PLS model.

Variables	R-square	R-square adjusted
X_2_: Group average gaze times	0.750	0.738
Y_1_: Group decision-making quality	0.060	−0.034
Y_2_: Group decision-making consensus degree	0.655	0.639
Y_3_: Group decision-making efficiency	0.758	0.747
Y_4_: Group decision-making satisfaction	0.791	0.769
Y_5_: Group decision-making acceptability	0.792	0.782

Since group average fixation count (X_2_) has been adjusted to be a mediator variable between X_1_ and Y_4_, its R^2^ represents the explanatory power of the independent variable X_1_ on the variance of X2. The *R*^2^-value is 0.750, and even after adjustment, the R2 adjusted remains at 0.738. This indicates that the variable has high stability and explanatory power in the model and can effectively predict its own variance.

In the fitting of the dependent variables, group decision satisfaction (Y_4_) and group decision acceptability (Y_5_) have the highest *R*^2^-values, at 0.791 and 0.792, respectively, and the R^2^ adjusted values also remain high at 0.769 and 0.782, respectively. This indicates that the model has strong explanatory power for these two dependent variables, effectively predicting group decision satisfaction and acceptability.

Next, group decision efficiency (Y_3_) and group decision consensus degree (Y_2_) have relatively high *R*^2^-values, at 0.758 and 0.655, respectively, with R^2^ adjusted values of 0.747 and 0.639. This suggests that the model can effectively explain and predict group decision efficiency and the degree of consensus.

Lastly, group decision quality (Y_1_) has a low *R*^2^-value of 0.060, with an adjusted R^2^ of −0.034, indicating that the current model has weak explanatory power for group decision quality. This may imply that other important factors affecting group decision quality have not been captured by the model, or that the relationship between the existing independent variables and group decision quality is more complex.

The model’s Variance Inflation Factor (VIF) scores are shown in Table 10. VIF scores are used to detect multicollinearity issues, where a VIF greater than 10 typically indicates the presence of multicollinearity. PLS-SEM reduces correlations between variables by constructing components that are linear combinations of the original variables, capturing most of the variance among them. Therefore, even in the presence of multicollinearity, PLS-SEM can provide stable estimates. In this study, all variables had VIF scores below 10, indicating that there is no significant multicollinearity issue among the variable combinations constructed in the PLS-SEM model.

**Table 10:** VIF scores of PLS model.

Variables	VIF
X_1_	1.000
X_2_	1.000
X_3_	1.000
X_4_	1.000
Y_1_ _obj	1.019
Y_1__sub	1.019
Y_2__obj	1.075
Y_2__sub	1.075
Y_3__obj	1.010
Y_3__sub	1.010
Y_4__sub	1.000
Y_5__sub	1.000

The path coefficient scores of the model are shown in Table 11. Path coefficient analysis reveals that, for the mediator variable group average fixation count (X_2_), the influence path “group average fixation duration (X_1_) - > group average fixation count (X_2_) - > group decision satisfaction (Y_4_)” is significant. However, for the mediator variable group decision quality (Y_1_), only the paths “group decision quality (Y_1_) - > group decision satisfaction (Y_4_) - > group decision acceptability (Y_5_)” and “group decision quality (Y_1_) - > group decision efficiency (Y_3_)” is significant.

**Table 11:** PLS model path coefficients.

Variables	Original sample (O)	Sample mean (M)	Standard deviation (STDEV)	T statistics (|O/STDEV|)	*p*-values
X_1_- > X_2_	0.866	0.862	0.047	18.261	0.000
X_2_ - > Y_4_	−0.230	−0.241	0.112	2.048	0.041
X_3_ - > Y_1_	0.112	0.099	0.180	0.621	0.534
X_4_ - > Y_1_	0.201	0.196	0.147	1.362	0.173
Y_1_- > Y_3_	0.871	0.845	0.103	8.452	0.000
Y_1_- > Y_4_	0.849	0.838	0.091	9.299	0.000
Y_3_ - > Y_2_	0.810	0.741	0.280	2.894	0.004
Y_4_ - > Y_5_	0.890	0.869	0.073	12.225	0.000

## 6 Experimental discussion and analysis

### 6.1 The effect of attention allocation on group decision-making performance

This study employs the Partial Least Squares Structural Equation Modeling (PLS-SEM) analysis method to explore the relationship between the visual attention allocation to technical-focused learning materials (four independent variables: group average fixation time ratio on HCN, SE, AD, EE, DWG—X_1_, group average fixation count—X_2_, group primary trajectory similarity—X_3_, and group fixation trajectory—X_4_) and group decision-making performance (five dependent variables: group decision quality—Y_1_, the degree of decision consensus—Y_2_, group decision efficiency—Y_3_, satisfaction with group decision outcomes—Y_4_, and decision acceptability—Y_5_). Through PLS-SEM analysis, both direct and indirect relationships among multiple variables can be assessed, providing deeper insights into the factors influencing group decision-making performance.

According to the PLS-SEM regression analysis model shown in Figure 6, it is evident that the visual attention allocation to different areas of interest (technical focus) in the decision-making materials primarily influences interdisciplinary group decision-making performance through two key indicators: group average fixation time and group average fixation count. These indicators directly affect participant satisfaction and the acceptability of group decisions, while indirectly influencing the quality of group decisions. Specifically, participants allocated visual attention to the HCN, SE, AD, and EE regions, which are closely related to the structure and function of core components in engineering design. Among these, the interdisciplinary technical focus area (HCN) received the highest proportion of visual attention.

Participants in the interdisciplinary technical focus areas exhibited higher average fixation time and fixation count, indicating that these areas contained critical information for the decision-making process or posed certain difficulties in understanding the technical focus. This finding is consistent with previous research, which shows that the most important conceptual nodes in design semantic networks are often interdisciplinary technical focus (Feng and Chen, 2022). In this experiment, these technical focal points served as key information within the decision-making materials, capturing participants’ visual attention and facilitating the construction of interdisciplinary cognitive structures and the ability to solve interdisciplinary engineering decision problems. The following four conclusions were drawn:

1. Group decision-making teams exhibited high levels of attention in areas related to “critical decision-making engineering semantics.”

2. Groups that spent more time focusing on the “critical decision-making engineering semantics” areas tended to demonstrate higher decision-making quality without external cues.

3. Although group similarity intuitively has a significant impact on decision performance, it is not statistically significant.

4. Participants from diverse disciplinary backgrounds often prioritize design terms and parameters relevant to their own fields, while overlooking interdisciplinary considerations. For example, decision-makers with expertise in structural engineering tend to emphasize aspects such as structural integrity and vibration dynamics. Those with an environmental engineering background focus on passenger comfort, including factors like temperature control and noise isolation. Similarly, decision-makers with an aesthetics background prioritize design elements that align with the ship type and brand image. However, an overemphasis on single-discipline perspectives can compromise decision quality or prolong discussions, ultimately reducing decision-making efficiency.

The path analysis results of the PLS-SEM model provide data support for the above conclusion 1 and conclusion 2:

1. The average group fixation duration positively influences the average group fixation count.

2. The average group fixation count positively impacts participant satisfaction and the acceptability of group decisions.

All these findings highlight the importance of participants’ deep understanding and consistent focus on interdisciplinary technical focal points in achieving high-quality, efficient group decision-making in interdisciplinary contexts.

In addition, participants’ allocation of visual attention to the drawing area (DWG) within the interdisciplinary decision-making materials had a negative impact on group decision-making performance. Specifically, excessive focus on the DWG area significantly reduced decision-making efficiency and the acceptability of group decisions. This may be due to the abstract nature of the information in the DWG area, which is less straightforward in conveying meaning compared to the text regions. This abstraction hindered participants’ understanding of design principles and methods, making it difficult for the group to differentiate between alternative solutions. As a result, it negatively affected the construction of interdisciplinary cognitive structures, leading to a decline in group decision-making efficiency.

### 6.2 Practical recommendations in engineering product design

In engineering product design, effective interdisciplinary group decision-making is essential for fostering innovation and practicality. This study, using eye-tracking technology, analyzed how attention allocation affects decision performance through technical-focused materials, offering data-driven insights. Based on the findings, the following recommendations are proposed:

1. *Optimize decision materials:* Enhance the layout and visual presentation of decision materials to direct attention to key information areas, especially technical focal points, using bold fonts, highlights, or color coding.
2. *Strengthen interdisciplinary training:* Provide interdisciplinary training to improve understanding across disciplines. Regularly assess cognitive abilities such as attention and memory, offering targeted support to enhance decision-making integration.
3. *Implement consensus-driven processes:* Develop processes that promote constructive discussions and multiple rounds of decision-making to ensure all viewpoints are considered, improving decision quality and comprehensiveness.

## 7 Conclusion and future work

This study presents an innovative approach to analyzing group decision-making performance in engineering design using eye-tracking technology. It quantitatively examines how decision-makers’ visual attention allocation impacts decision performance, identifying average fixation time and counting as key factors influencing satisfaction and acceptability. These findings contribute to new theoretical insights and offer practical guidance for achieving high-quality, efficient decisions in engineering product design.

The key innovations of this study are: (1) *Employing eye-tracking technology* to investigate visual attention dynamics in interdisciplinary group decision-making contexts; (2) *Developing a novel performance evaluation framework* that connects attention allocation mechanisms to decision-making outcomes using metrics such as average fixation count and gaze trajectory similarity; and (3) *Demonstrating the method’s practical applicability* through a real-world case study focused on the design of cabins for Chinese cruise ships.

This study offers several key contributions: (1) It introduces a multi-attribute group decision-making paradigm, integrating engineering knowledge with cognitive science to enhance communication and decision-making efficiency across disciplines. (2) By quantifying visual attention allocation, it employs PLS-SEM analysis to develop a performance evaluation model, revealing how attention to technical foci impacts decision outcomes. (3) The method’s application in ship cabin design validates its effectiveness in improving decision quality, efficiency, and satisfaction. Additionally, the study provides strategies to overcome interdisciplinary decision-making challenges, including optimizing decision materials and fostering consensus-driven processes.

The proposed model has some limitations: (1) The influence paths related to gaze trajectory similarity show weak statistical performance, indicating the need for improved interpretability and predictive accuracy. (2) The mechanisms of intermediate or latent variables remain unclear, requiring further experiments and theoretical analysis in future research.

To address the issues, future research can be improved in the following ways:

1. *Expanding the sample size*: Increasing the sample size will enhance the statistical power of the model, which may improve the significance of the loadings.
2. *Exploring new measurement indicators:* Introducing new metrics or methods, such as psychophysiological indicators, could more comprehensively capture the multidimensional characteristics of group decision-making performance.
3. *Considering potential mediating variables:* Exploring whether potential mediating variables, such as team trust and communication quality, influence the relationship between independent and dependent variables.
4. *Multi-wave data analysis:* Utilizing multi-wave data to analyze the dynamic changes in visual attention allocation during the decision-making process may help uncover more complex decision mechanisms.

This paper demonstrates the validity of the experimental analysis results in the actual interdisciplinary decision-making process through experimental validation in the case of cabin design in a shipbuilding company. Although the problem of ship cabin design only covers the knowledge of ship structure, environmental engineering and aesthetics, the knowledge distribution of the disciplines to solve the problem is relatively even, and there is no disciplinary barrier, so it can be inferred that the experimental method and the experimental conclusions are also applicable to the design of products in other fields of engineering, such as aerospace, automobile manufacturing, biomedical engineering, urban planning, and so on. In addition, because the design case comes from engineering design practice, the decision-making material, decision-making process, decision-making personnel ratio are basically consistent with the engineering practice, and has been recognized by the experts of a shipbuilding company, it can be applied to the real-time decision support system engineering company.

In summary, this study offers key insights for engineering design decision-making: organizations should optimize decision materials, enhance interdisciplinary training, and adopt consensus-driven processes. As eye-tracking technology advances and data quality improves, future research will focus on refining model parameters to better handle large datasets and enhance scalability. Further validation in fields like aerospace and automotive design is expected. Additionally, future studies will explore the impact of mediating variables, such as team trust and communication quality, and apply multi-wave data analysis to uncover dynamic decision-making mechanisms, providing more comprehensive theoretical and practical support.

## Data Availability

The original contributions presented in the study are included in the article. Further inquiries can be directed to the corresponding author/s.
